# Synthesis and Properties of In-Situ Bulk High Impact Polystyrene Toughened by High *cis*-1,4 Polybutadiene

**DOI:** 10.3390/polym11050791

**Published:** 2019-05-02

**Authors:** Feng Wang, Li Chang, Yanming Hu, Guangfeng Wu, Heng Liu

**Affiliations:** 1 School of Chemical Engineering, Changchun University of Technology, Changchun 130012, China; wangfeng@ccut.edu.cn; 2 CAS Key Laboratory of High-Performance Synthetic Rubber and its Composite Materials, Changchun Institute of Applied Chemistry, Chinese Academy of Sciences, 5625 Renmin Street, Changchun 130022, China; changli@163.com (L.C.); ymhu@ciac.ac.cn (Y.H.)

**Keywords:** high impact polystyrene, in-situ bulk polymerization, morphology, polybutadiene, radical polymerization

## Abstract

A series of high impact polystyrenes (HIPS) were successfully prepared by in-situ bulk polymerization. Selective polymerization of butadiene (Bd) in styrene (St) was achieved by using a neodymium bis(2-ethylhexanol)phosphonate/diisobutyl aluminum hydride/diethylaluminum chloride (Nd(P_507_)_3_/Al(*i*-Bu)_2_H/AlEt_2_Cl) catalyst, affording a prepolymer with high *cis*-1,4 content of 96.6% and extremely low styrene content of 1.01%. The prepolymer solution was further subjected to the radical polymerization of styrene to produce HIPS. The impact strength of HIPS increased from 48.5 to 166.2 J/m, with an increase of rubber content from 5% to 15%, whereas the tensile strength at break decreased from 17 to 12.5 MPa. Moreover, the impact strength and the tensile strength at break of HIPS increased from 72.4 J/m to 120.6 J/m and 13.2 to 16 MPa, respectively, as the initiator concentration decreased from 0.05% to 0.01%. HIPS showed the highest impact strength of 84.8 J·m^−1^ and displayed well “salami” morphology when using a trifunctional 3,6,9-triethyl-3,6,9-trimethyl-1,4,7-triperoxonane initiator.

## 1. Introduction

The incorporation of a rubbery phase into glassy polymers is recognized as an effective method to significantly improve the toughness of the materials. High-impact polystyrene (HIPS) is a common type of rubber toughened polymeric material and displays good balance between rigidity and elasticity. HIPS is employed in various fields including automobiles, packaging, electrical instruments, and so forth [1,2]. In general, HIPS is produced by bulk polymerization or bulk-suspension polymerization of styrene in the presence of rubber, such as polybutadiene (PB), styrene-butadiene copolymer, and ethylene-propylene copolymer. In special, the high impact property of HIPS at a low temperature can be obtained when high *cis*-1,4 polybutadiene is used as a toughening agent [3]. 

In recent decades, a so-called in-situ bulk polymerization process for the synthesis of high impact resins has been reported [4]. In this process, polybutadiene prepolymer solution in styrene is prepared, and then the unreacted styrene or styrene/acrylonitrile is polymerized by a radical polymerization. Compared to the typical bulk polymerization or bulk-suspension polymerization [5,6,7], the cost of such technology remarkably reduces because the rubber does not need isolation from the polymerization system and further dissolving in the corresponding monomers and/or solvent. To date, a variety of catalysts based on lithium, transition metals (cobalt, nickel, and titanium), and rare earth have been employed in such procedures. Among them, catalysts based on rare earth metals, in particular neodymium (Nd), have attracted much attention for the polymerization of conjugated dienes due to their high catalytic activity, high *cis*-1,4 stereospecificity, and extremely low tendency of gel formation [8]. In our previous study, it was found that the Nd-based catalyst can selectively polymerize butadiene in styrene, and subsequently the acrylonitrile butadiene styrene (ABS) resin with high impact strength was synthesized through the in-situ bulk polymerization method [9]. On the other hand, due to the excellent elasticity and low temperature resistance of high *cis*-1,4 polybutadiene, the prepared HIPS with high *cis*-1,4 polybutadiene as a toughening agent exhibits high impact resistance even at a low temperature [2,10]. Moreover, the residue of the Nd-based catalyst does not affect the mechanical properties of the produced polymers [8]. 

HIPS is a multicomponent and multiphase material with continuous phase of polystyrene (PS) matrix and dispersed phase of rubber particles with PS occlusions. The rubber particles generally exhibit two kinds of morphologies. The “salami” morphology, with particles of 1–3 μm, is generally prepared by dissolution of polybutadiene polymer into pure styrene monomer as solvent and then the polymerization of styrene in the presence of a initiator. In “salami” morphology, each of the larger rubber particles contains multiple PS occlusions. The “core-shell” morphology with general sub-micrometer particles, however, is formed by melt blending of PS with styrene/butadiene copolymer, and in such as morphology, each of the smaller rubber particles contains a single PS occlusion [11,12]. Choi and Sharma have studied the effects of microstructure and morphology of HIPS on their mechanical properties [13,14]. To the best of our knowledge, however, there are few reports related to the study on such polymerization method of HIPS prepared by in-situ bulk polymerization. 

In the present study, we reported the synthesis and properties of HIPS through an in-situ bulk polymerization approach. An Nd(P_507_)_3_/Al(*i*-Bu)_2_H/AlEt_2_Cl catalyst was used for the selective polymerization of butadiene in styrene to produce a solution of butadiene prepolymer. The resultant prepolymer solution was directly used to prepare HIPS. Furthermore, the effects of the prepolymer content and initiator type and concentration on the properties of HIPS were investigated in detail.

## 2. Experimental

### 2.1. Materials

Neodymium bis(2-ethylhexanol)phosphonate (Nd(P_507_)_3_) was donated by the Changchun Institute of Applied Chemistry. Al(*i*-Bu)_2_H and AlEt_2_Cl (Nanjing Tonglian Chemical Co., Nanjing, China, 1.0 M solution in hexane) were used as received. Benzoyl peroxide (BPO, Shanghai Zhongli Chemical Co., Shanghai, China) was purified by recrystallization. 1,1-di-(*tert*-butylperoxy)-3,3,5-trimethylcyclohexane (DP275B, Jiangsu Qiangsheng Chemical Co., Jiangsu, China), 3,6,9-triethyl-3,6,9-trimethyl-1,4,7-triperoxonane (TETMTPA, J&K Chemical Co., USA), and 2,2-bis(4,4-di-(*tert*-butyl-peroxy-cyclohexyl)propane) (TBPP, J&K Chemical Co., USA) were used as received. Butadiene (PetroChina Jinzhou Petrochemical Branch, Liaoning, China) was purified by passing through the columns of sodium hydroxide and molecular sieve before use. Styrene and ethylbenzene (Daqing Petrochemical Co., Heilongjiang, China) were purified by the standard method. 

### 2.2. Preparation of Catalyst

The vessel was purged with dry nitrogen, and then Nd(P_507_)_3_ (0.26 × 10^−3^ mol) and Al(*i*-Bu)_2_H (2.0 ml, 2.0 M) was added. After the mixture was left for 10 min at 30 °C, AlEt_2_Cl (0.27 ml, 2.0 M) was added, and the catalyst solution was aged for another 30 min. Then, the rare earth catalyst was obtained.

### 2.3. Preparation of Butadiene Prepolymer Solution 

A polymerization vessel was sequentially charged with 180 g of styrene, 20 g of 1,3-butadiene and the prepared catalyst. Polymerization was carried out at 50 °C for 4 h and quenched by adding small amount of isopropanol containing antioxidant 2,6-di-*tert*-butyl-4-methylphenol (1.0 wt %) as a stabilizer. 

### 2.4. Synthesis of HIPS Resins

After addition of the initiator and small amount of ethylbenzene into the butadiene prepolymer solution, the polymerization was carried out at 90, 106, 110, and 135 °C, respectively, corresponding to the decomposition temperature of the initiators. After 5 h, the polymerization temperature increased to 180 °C, and the reaction was kept for another 2 h. The produced mixture was dissolved in chloroform and precipitated in ethanol three times, and cut into small pieces and dried under a vacuum at 40 °C for 24 h. 

### 2.5. Measurements and Characterization

^1^H NMR spectra were recorded with an AVANCE400 spectrometer in CDCl_3_ at room temperature. IR spectra of polybutadienes were examined by a Nicolet 20DXB spectrophotometer. Film samples were prepared on a KBr disc by casting CHCl_3_ solution of polymer. The proportion of *cis*-1,4, *trans*-1,4 and 1,2-vinyl units was determined from the absorption bands at 727, 965, and 911 cm^−1^, respectively. The molecular weights (*M*_w_) and molecular weight distributions (*M*_w_/*M*_n_) of polymers were estimated by gel permeation chromatography equipped with a Waters 515 HPLC pump and a Waters 2414 differential refractometer using tetrahydrofuran (THF) as eluent at a flow rate of 1.0 mL/min, calibrated with polystyrene standard.

### 2.6. Determination of Grafting Parameters

0.150 g of sample was dissolved in 30.0 g of acetone and refluxed for 8 h. The mixture was centrifuged at 6000 rpm in a TG-16 ultracentrifuge for half an hour three times. The insoluble precipitate (PB and PS_graft_) was isolated from the soluble fraction, and the soluble fraction (PS_free_) was precipitated and filtered from ethanol. Both of them were dried under vacuum at 40 °C to constant weight. Grafting degree (GD) and rubber phase volume fraction (RPVF) were calculated as follows:

(1) $$\begin{array}{l} GD=\frac{(PB+PS_{graft})-PB}{PB}\times 100\% \end{array}$$

(2) $$RPVF=\frac{PB+PS_{graft}}{PB+PS_{graft}+PS_{free}}\times 100\%$$

### 2.7. Notched IZOD Impact Testing

The notched specimens were prepared by Model 2 Injection Test Sample Molding Apparatus (Ray-Ran, UK) and placed at 23 °C for at least 48 h before testing. The dimensions of the specimen were 80 mm × 10 mm × 4 mm with a notch tip of 2 mm depth. The tests were performed on a CEAST RESILE IMPACTOR (Italy) at 23 °C according to ASTM D-256. An average of five specimens was taken for each sample. 

### 2.8. Tensile Testing

Dumbbell specimens measuring 4.0 mm × 2.1 mm were shaped from the molded sheets and used for tensile tests. The tests were carried out at a crosshead speed of 5 mm/min in the temperature of 23 °C following ASTM D-638. An average of five specimens was taken for each sample.

### 2.9. Transmission Electron Microscopy (TEM)

The morphology of HIPS was observed with a FEI TECNAI20 TEM (USA), using a voltage of 200 kV. Ultramicrotomed sections were firstly prepared by a LEICA ULTRACUT UCT (Switzerland), and then stained with OsO_4_ for 24 h before observation. 

## 3. Results and Discussion

### 3.1. Polymerization of Butadiene

Polymerization of butadiene was carried out in styrene by using Nd(P_507_)_3_/Al (*i*-Bu)_2_H/AlEt_2_Cl catalyst, and the results are listed in Table 1. The catalyst exhibited high activity and high *cis*-1,4 selectivity towards the polymerization of butadiene. The conversion of butadiene was quantitative, whereas only 0.5% styrene was polymerized. Determined by Gel Permeation Chromatography (GPC, Figure S1), the resultant prepolymer of butadiene had a molecular weight of 17.7 × 10^4^ and a molecular weight distribution of 3.05.

**Figure 1:**
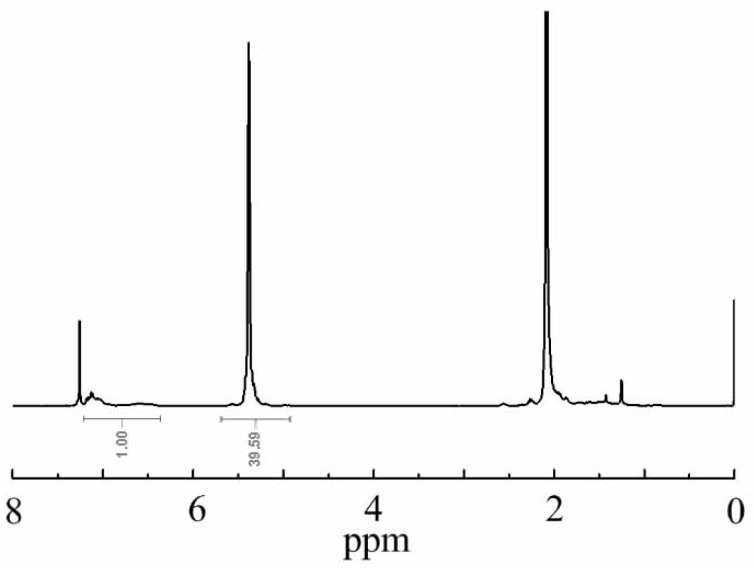
^1^H NMR spectrum of polybutadiene in CDCl_3_.

It is well known that high *cis*-1,4 polydienes can be obtained with Nd-based catalysts [8,15]. The microstructure of the polymer was determined by ^1^H NMR and IR spectra. As shown in Figure 1, the weak signals at 6.5–7.2 ppm assigned to St units indicate that the prepolymer contained relatively low St content of 1.00%. The sharp signal at 5.38 ppm is attributed to the 1,4 units of butadiene. Because ^1^H NMR cannot be used to distinguish the *cis*-1,4 and *trans*-1,4 units of polybutadiene, the IR spectrum was further used to determine the contents of the *cis*-*trans* 1,4-isomers.

**Figure 2:**
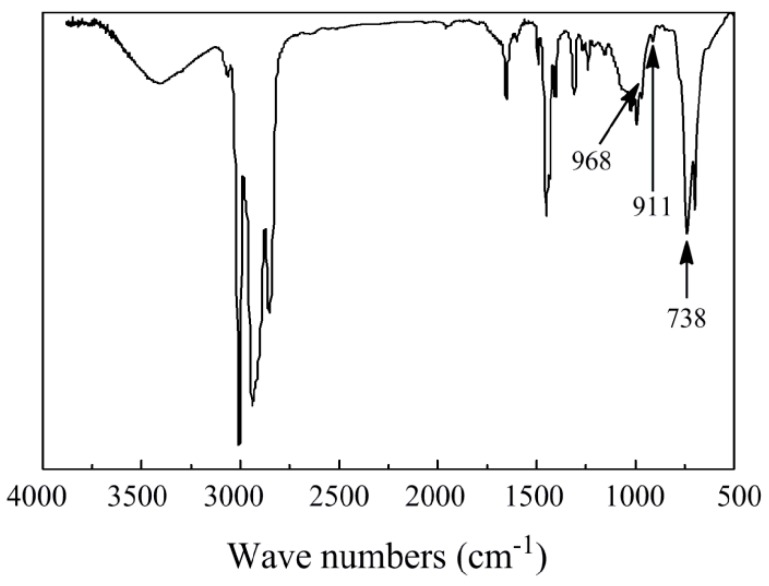
The FTIR spectrum of polybutadiene.

Figure 2 is the IR spectrum of the same polymer. The absorption bands at 738, 911, and 968 cm^−1^ are characteristic of *cis*-1,4, 1,2, and *trans*-1,4 units, respectively. Calculated on the basis of previous literature [16], the prepolymer had 96.6% of *cis*-1,4 units, 2.6% of *trans*-1,4 units, and 0.8% of 1,2-vinyl units. It has been reported that high impact property at low temperature could be obtained with high *cis*-1,4 polybutadiene as a toughening agent [2,10]. Thus, the impact property of HIPS by using the above-synthesized polybutadiene as a toughening agent was examined.

### 3.2. Effect of Bd Prepolymer Content on the Morphologies and Properties of HIPS

HIPS was synthesized by the in situ bulk polymerization of styrene with TETMTPA as initiator in the prepolymer solution. The effect of the rubber content on the impact strength of HIPS was examined, and the results are summarized in Table 2. The *M*_w_ of HIPS hardly changed and stayed around 22.0 × 10^4^ at the rubber content from 5% to 10%. When the rubber content increased to 15%, the *M*_w_ sharply decreased to 17.7 × 10^4^, and the molecular weight distribution increased to 2.24. In addition, the amount of 1,2 structure of polybutadiene, which is conducive to the grafting reaction, increased with the increase of the rubber content, and a higher RPVF content of HIPS was obtained.

The impact strength of HIPS tended to increase with increasing rubber content. For instance, the impact strength of HIPS at the rubber content of 15% was 166.2 J/m, which was about four times as large as that at the rubber content of 5%, where the lower impact strength might be due to the formation of relatively small rubber particles (<500 nm) at low rubber content of 5% [17,18,19]. As the rubber content increased from 5% to 10%, the tensile strength at break hardly changed and stayed about 17 MPa. When it increased to 15%, the tensile strength at break significantly reduced to 12.5 MPa. Similar results were observed in the previous studies [5,20].

**Figure 3:**
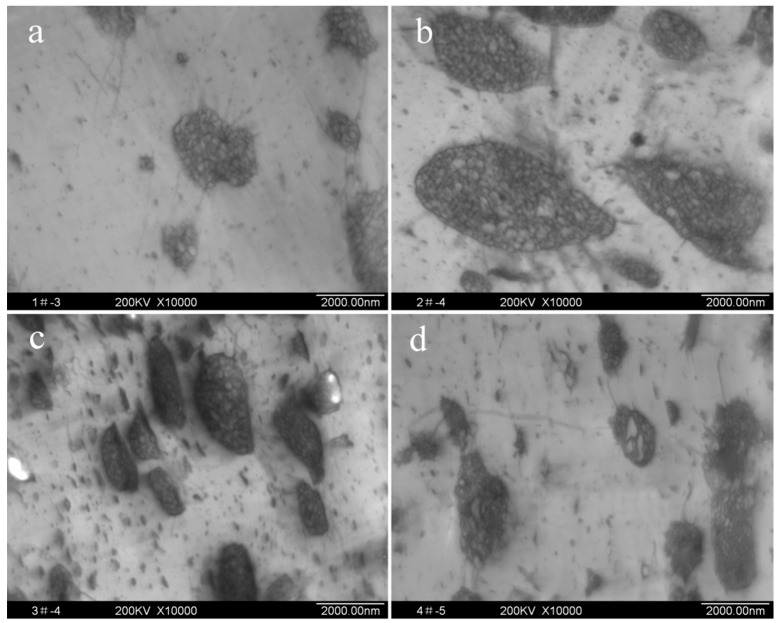
TEM micrographs of HIPS containing different rubber contents. (**a**) 5.0 wt %; (**b**) 7.5 wt %; (**c**) 10.0 wt %; (**d**) 15.0 wt %.

Figure 3 shows the effect of rubber content on the particle morphology. The white region represents the resin phase (continuous phase) and the black region represents the rubbery phase (dispersed phase). The “salami” morphologies were clearly observed in the HIPS containing 5.0–15.0 wt % of rubber content. As the rubber content increased, the occlusions become larger and could not be enclosed well by the rubber layers, and the mass ratio of PS occlusions to PB of each particle decreased. This can be explained as follows: the grafting reaction of St and PB including external grafting occurs on the surface of the rubber particles; and internal grafting forms the PS occlusions inside the rubber particles [21]. Thus, external grafting and internal grafting affect particle distribution in the PS continuous phase and internal structure of particles, respectively. In the higher rubber content, PB may form continuous network structure, which is harmful to the grafting reaction. Therefore, although the total fraction of St grafted on PB increased with increasing the rubber content, the amount of St grafted per unit of PB actually decreased [22].

### 3.3. Effect of Initiator Concentration on the Morphologies and Properties of HIPS 

As can be seen from Table 3, the *M*_w_ of PS was around 23.0 × 10^4^ at the initiator concentration of 0.01–0.03%. At the higher initiator concentration of 0.05%, the *M*_w_ decreased to 20.7 × 10^4^. Both RPVF and GD increased with increasing the initiator concentration, while the impact strength of HIPS decreased. This might be due to the uniform dispersion and higher elasticity of rubber particles in HIPS at the lower initiator concentration [18]. Moreover, the tensile strength at break kept constant at the initiator concentration from 0.01% to 0.03%, and then decreased to 13.2 MPa at initiator concentration at 0.05%. That is, HIPS with higher RPVF and larger rubber particles usually showed the lower tensile strength. 

**Figure 4:**
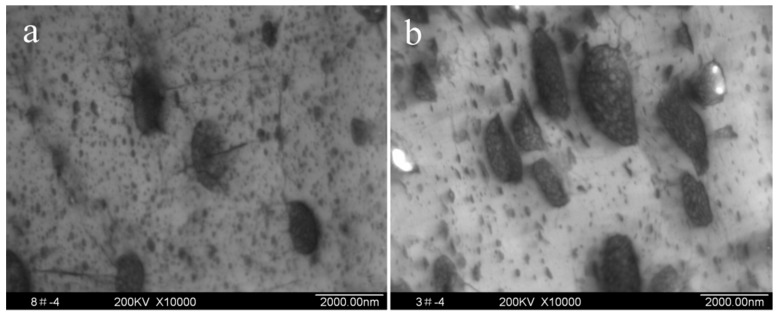
TEM micrographs of HIPS at different initiator concentrations. (**a**) 0.01%; (**b**) 0.03%.

TEM micrographs of HIPS obtained at different initiator concentrations are shown in Figure 4. The rubber particle size in HIPS increased with the concentration of initiator increasing. This result was consistent with the previous reports [12,17]. This result might be due to the higher grafting degree attained at higher initiator concentrations, leading to the formation of larger sized particles. On the other hand, well-distributed particles were observed in the HIPS produced at a low initiator concentration of 0.01%, while the dispersion of particles became worse at the initiator concentration of 0.03%.

### 3.4. Effect of Initiator Type on the Morphologies and Properties of HIPS

HIPS were prepared with four kinds of initiators, i.e., monofunctional BPO, bifunctional DP275B, trifunctional TETMTPA, and tetrafunctional TBPP at an equivalent functionality. Due to a higher number of functional sites per molecule, the multifunctional initiator can simultaneously increase polymerization rate and polymer molecular weight [23,24]. As shown in Table 4, the polymers obtained with DP275B and TBPP had higher *M*_w_ (34.3 × 10^4^ and 27.3 × 10^4^, respectively) than that with BPO (*M*_w_ = 22.2 × 10^4^). The *M*_w_/*M*_n_ of HIPS was independent of the initiator type and kept around 1.75. 

**Figure 5:**
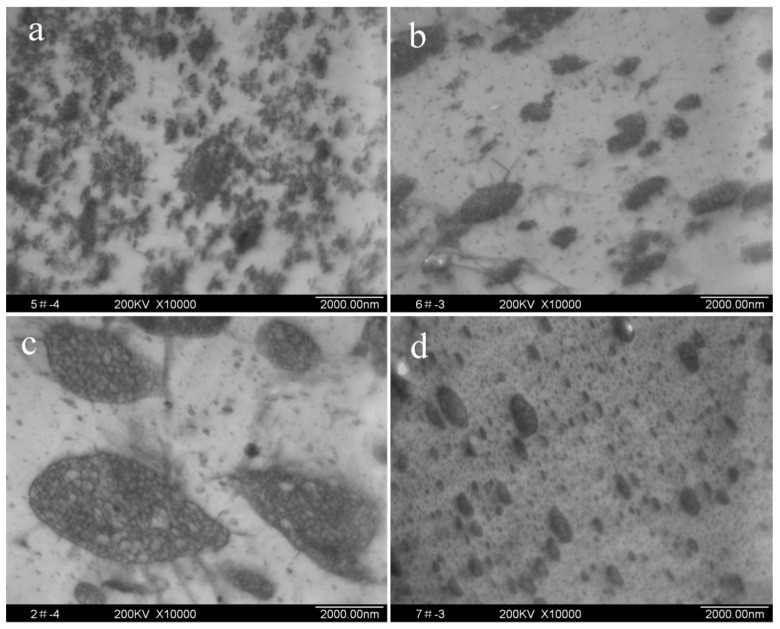
TEM micrographs of HIPS prepared by different initiator types. (**a**) BPO; (**b**) DP275B; (**c**) TETMTPA; (**d**) TBPP.

The morphologies of HIPS were examined by TEM (Figure 5). It is clear that the particles of HIPS obtained with BPO are small and irregular, and the existence of elliptic particles and “salami” morphologies is observed in HIPS obtained with TETMTPA. Although there were a large number of elliptic particles in HIPS obtained with DP275B and DPBB, “salami” morphologies are not observed clearly. The particles of HIPS obtained with DPBB are smaller than those with DP275B.

In fact, HIPS showed different mechanical properties, especially impact strength, with different initiators. For example, HIPS with TETMTPA as an initiator showed the highest impact strength of 84.8 J/m, which was about 6 times as large as that with TBPP as an initiator, as shown in Table 4. Correspondingly the higher impact strength was due to the larger rubber particles with “salami” morphology. 

## 4. Conclusions

A series of HIPS were prepared via in-situ bulk polymerization. Firstly polymerization of butadiene in styrene using Nd(P_507_)_3_/Al(*i*-Bu)_2_H/AlEt_2_Cl as a catalyst was carried out and formed the butadiene prepolymer solution. The produced butadiene prepoplymer had high *cis*-1,4-butadiene, with a content of 96.6%, and relatively low St content of 1.01%. The resultant prepolymer solution was directly used to produce HIPS by a radical polymerization with different initiators. At the rubber content of 5.0–15.0 wt %, the “salami” morphologies were well obtained. The impact strength of HIPS increased from 48.5 J/m to 166.2 J/m with the increase of rubber contents, whereas the tensile strength at break decreased from 17.0 to 12.5 MPa. Moreover, the particle size of HIPS increased with increasing concentration of the initiator, but the impact strength and the tensile strength decreased from 120.6 J/m and 16.0 MPa to 72.4 J/m and 13.2 MPa, respectively. Compared to other initiators, HIPS using TETMTPA as an initiator showed a “salami” morphology and the highest impact strength at 84.8 J/m.

## Figures and Tables

**Table 1:** Polymerization of butadiene with Nd(P_507_)_3_/Al(*i*-Bu)_2_H/AlEt_2_Cl catalyst in styrene ^a^.

Conversion (%)		St Content ^b^ (mol %)	Microstructure ^c^ (%)			*M*_w_ × 10^−4 d^	*M*_w_/*M*_n_^d^
Bd	St		*cis*-1,4	*trans*-1,4-	1,2-		
100	0.5	1.01	96.6	2.6	0.8	17.7	3.05

^a^ Polymerization at 50 °C for 4 h, [Nd]/[Bd] = 4.3 × 10^−4^, [Al]/[Nd] = 15.0, [Cl]/[Nd] = 2.0, St/Bd = 9.0 (weight ratio). ^b^ Determined by ^1^H NMR. ^c^ Determined by FTIR. ^d^ Measured by GPC calibrated with polystyrene. *M*_w_: molecular weight.

**Table 2:** Characteristics and properties of high impact polystyrenes (HIPS) containing different rubber contents ^a^.

PB (wt %)	RPVF (%)	GD (%)	PS		Mechanical Properties	
			*M*_w_ × 10^−4 b^	*M*_w_/*M*_n_^b^	Impact Strength (J/m)	Tensile Strength at Break (MPa)
5.0	10.0	78.1	21.8	1.76	48.5	16.5
7.5	14.3	84.7	20.8	1.86	84.8	16.8
10.0	19.6	85.0	22.9	1.87	86.0	16.7
15.0	35.8	117.9	17.4	2.24	166.2	12.5

^a^ Conditions: T = 135 °C, TETMTPA = 0.03 wt %, r = 200 rpm, EB = 20 wt %. ^b^ Measured by GPC calibrated with standard polystyrenes. RPVF: rubber phase volume fraction; PB: polybutadiene; GD: grafting degree; PS: polystyrene; TETMTPA: 3,6,9-triethyl-3,6,9-trimethyl-1,4,7-triperoxonane.

**Table 3:** Effect of TETMTPA concentration on the properties of HIPS ^a^.

TETMTPA (wt %)	RPVF (%)	GD (%)	PS		Mechanical Properties	
			*M*_w_ × 10^−4 b^	*M*_w_/*M*_n_^b^	Impact Strength (J/m)	Tensile Strengthat Break (MPa)
0.01	17.2	50.4	22.6	1.85	120.6	16.1
0.02	18.3	68.2	22.6	1.89	107.4	16.0
0.03	19.6	85.0	22.9	1.87	86.0	16.7
0.05	23.8	131.4	20.7	1.80	72.4	13.2

^a^ Conditions: T = 135 °C, PB = 10 wt %, r = 200 rpm, EB = 20 wt %. ^b^ Measured by GPC calibrated with standard polystyrenes.

**Table 4:** Effect of different initiators on the properties of HIPS ^a^.

Initiator		RPVF (%)	GD (%)	PS		Mechanical Properties	
Type	Content (wt %)			*M*_w_ × 10^−4 b^	*M*_w_/*M*_n_^b^	Impact Strength (J/m)	Tensile Strengthat Break (MPa)
BPO	0.090	22.1	92.1	22.2	1.72	19.2	18.3
DP275B	0.045	28.2	167.1	34.3	1.70	45.8	16.0
TETMTPA	0.030	14.3	84.7	20.8	1.86	84.8	16.8
TBPP	0.023	36.8	195.6	27.3	1.69	13.5	20.9

^a^ Conditions: PB = 7.5 wt %, r = 200 rpm, EB = 20 wt %. ^b^ Measured by GPC calibrated with standard polystyrenes. BPO: benzoyl peroxide; DP275B: 1,1-di-(*tert*-butylperoxy)-3,3,5-trimethylcyclohexane; TBPP: 2,2-bis(4,4-di-(*tert*-butyl-peroxy-cyclohexyl)propane).

## Supplementary Materials

The following are available online at https://www.mdpi.com/2073-4360/11/5/791/s1, Figure S1: GPC curve for polybutadiene in Table 1.
