# Supplementary figures and images for: Synthesis and Properties of In-Situ Bulk High Impact Polystyrene Toughened by High cis-1,4 Polybutadiene
Source: Polymers (Basel). 2019 May 2;11(5):791. doi: 10.3390/polym11050791 (PMC6571887; doi:10.3390/polym11050791)

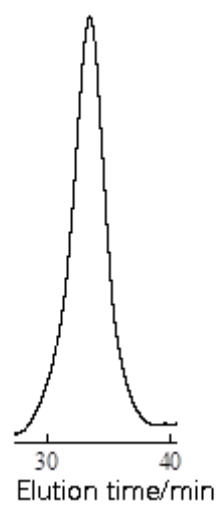

Figure S1. GPC curve for polybutadiene in Table 1.

Supplement: Supplementary file 1 [file polymers-11-00791-s001.pdf]
